# Regulatory T Cells Enhance Susceptibility to Experimental *Trypanosoma Congolense* Infection Independent of Mouse Genetic Background

**DOI:** 10.1371/journal.pntd.0001761

**Published:** 2012-07-31

**Authors:** Ifeoma Okwor, Chukwunonso Onyilagha, Shiby Kuriakose, Zhirong Mou, Ping Jia, Jude E. Uzonna

**Affiliations:** 1 Department of Medical Microbiology, Faculty of Medicine, University of Manitoba, Winnipeg, Manitoba, Canada; 2 Department of Immunology, Faculty of Medicine, University of Manitoba, Winnipeg, Manitoba, Canada; Institut Pasteur, France

## Abstract

**Background:**

BALB/c mice are highly susceptible while C57BL/6 are relatively resistant to experimental *Trypanosoma congolense* infection. Although regulatory T cells (Tregs) have been shown to regulate the pathogenesis of experimental *T. congolense* infection, their exact role remains controversial. We wished to determine whether Tregs contribute to distinct phenotypic outcomes in BALB/c and C57BL/6 mice and if so how they operate with respect to control of parasitemia and production of disease-exacerbating proinflammatory cytokines.

**Methodology/Findings:**

BALB/c and C57BL/6 mice were infected intraperitoneally (i.p) with 10^3^ *T. congolense* clone TC13 and both the kinetics of Tregs expansion and intracellular cytokine profiles in the spleens and livers were monitored directly *ex vivo* by flow cytometry. In some experiments, mice were injected with anti-CD25 mAb prior or post *T. congolense* infection or adoptively (by intravenous route) given highly enriched naïve CD25^+^ T lymphocytes prior to *T. congolense* infection and the inflammatory cytokine/chemokine levels and survival were monitored. In contrast to a transient and non significant increase in the percentages and absolute numbers of CD4^+^CD25^+^Foxp3^+^ T cells (Tregs) in C57BL/6 mouse spleens and livers, a significant increase in the percentage and absolute numbers of Tregs was observed in spleens of infected BALB/c mice. Ablation or increasing the number of CD25^+^ cells in the relatively resistant C57BL/6 mice by anti-CD25 mAb treatment or by adoptive transfer of CD25^+^ T cells, respectively, ameliorates or exacerbates parasitemia and production of proinflammatory cytokines.

**Conclusion:**

Collectively, our results show that regulatory T cells contribute to susceptibility in experimental murine trypanosomiasis in both the highly susceptible BALB/c and relatively resistant C57BL/6 mice.

## Author Summary

BALB/c mice are highly susceptible while C57BL/6 is relatively resistant to experimental *Trypanosoma congolense* infection. Acute death observed in infected BALB/c mice is usually associated with the excessive production of pro-inflammatory cytokines. Regulatory T cells (Tregs) have been shown to play a significant role in the pathogenesis of many diseases including those caused by parasites. However, the role of Tregs in the pathogenesis of *T. congolense* infection remains unclear. We were interested in addressing the following questions: Do Tregs contribute to the distinct phenotypic outcomes observed in *T. congolense*-infected BALB/c and C57BL/6 mice? If so, where and how do they operate with respect to parasitemia and cytokine response? By selectively altering the numbers of these cells either by targeted depletion with monoclonal antibody or adoptive transfer of highly enriched naïve CD25^+^ cells prior to infection, we show that Tregs impairs efficient parasite control and impacts on production of disease-exacerbating proinflammatory cytokines. Collectively, our findings suggest that Tregs contribute to enhanced susceptibility to experimental *T. congolense* infection in mice.

## Introduction

Tse-Tse-transmitted trypanosomiasis is a complex disease in both humans and animals caused by several species of the protozoan parasite *Trypanosoma* [1]. *Trypanosoma brucei gambiense* and *Trypanosoma brucei rhodensiense* cause disease in humans (sleeping sickness) while trypanosomiasis in animals (Nagana) is caused by *Trypanosoma congolense*, *Trypanosoma brucei brucei* and *Trypanosoma vivax*. It is estimated that human African trypanosomiasis (HAT) accounts for 1.6 million disability adjusted life years in sub-saharan Africa per year, thereby putting a huge burden on poor rural farmers [1]. Out of the three species of animal trypanosomiasis, *T. congolense* is the most important disease for livestock [2].

Some cattle breeds indigenous to West Africa (such as the Ndama) are relatively resistant to trypanosomiasis, whereas the European breeds, particularly of the Zebu background are highly susceptible. The immunologic mechanisms that regulate this difference in susceptibility are not clearly understood. Similarly, different strains of inbred mice show varying degree of susceptibility to experimental *T. congolense* infection. For example, BALB/c mice are highly susceptible while the C57BL/6 mice are relatively resistant to infection with *T. congolense* as measured by the ability to control parasitemia and survival period [3], [4]. Resistance is typically linked with the early production of interferon gamma (IFN-γ), nitric oxide and parasite-specific IgG2a antibodies, which are essential for parasite clearance [5], [6], [7]. However, the over production of IFN-γ as well as other proinflammatory cytokines, (particularly IL-1β, IL-6, IL-12 and TNF) contributes to disease and death in the highly susceptible BALB/c mice [8]. Indeed, IL-10, which has powerful anti-inflammatory properties, is critical for survival of mice infected with *T. congolense*. Blockade of IL-10 signaling by treatment with anti-IL-10R mAb leads to the elevation of serum levels of proinflammatory cytokines and acute death in otherwise relatively resistant C57BL/6 mice [8] and LTβR^−/−^ mice on C57BL/6 background [9].

Gershon et.al [10] reported the presence of thymic CD4^+^ lymphocyte populations capable of suppressing antigen-specific immune responses and these cells were later characterized as naturally occurring regulatory T cells (Tregs) [11]. Tregs constitutively express CD25 (interleukin IL-2 receptor α chain) and the transcription factor; forkhead box protein 3 (Foxp3) [12] and are mainly involved in the regulation and control of autoreactive T cells. In addition, Tregs have also been shown to influence the pathogenesis of several infectious diseases including bacteria [13] fungi [14] and parasites [15], [16]. However, the role of Tregs in the pathogenesis of African Trypanosomiasis is controversial. While a report indicates that Tregs play a crucial role in enhanced resistance [17] another report showed that they contribute to susceptibility to the infection [18]. This discrepancy could be related to differences in mouse strains (BALB/c versus C57BL/6) used in these experiments by the two different groups. Furthermore, these studies relied solely on antibody depletion of CD25^+^ cells and did not directly test the role of Tregs by adoptive transfer. Here, we asked whether Tregs contribute to the distinct disease outcomes observed in *T. congolense*-infected BALB/c and C57BL/6 mice. Our findings indicate that Tregs play pathogenic roles in African trypanosomiasis in both the relatively resistant and highly susceptible mice. The extent of this effect is highly variable, being more pronounced in the relatively resistant mice and only observable in the highly susceptible mice following an intradermal infection.

## Materials and Methods

### Ethics statement

All experimental protocols were approved by the Animal Care and Use Committee of University of Manitoba and all animals were housed and used according to the guidelines stipulated by the Canadian Council for Animal Care.

### Mice

Six to eight (6–8) weeks old female C57BL/6, BALB/c and outbred CD-1 mice were purchased from the University of Manitoba Central Animal Care Services (CACS) breeding facility. All mice were maintained in specific-pathogen free environment at the University of Manitoba CACS.

### Parasites and infection

Cryopreserved *Trypanosoma congolense*, variant antigenic type (VAT) TC13 were passaged in CD-1 mice as previously described [19]. Three days post-passage, mice were sacrificed and bloodstream forms of the parasites were isolated by DEAE-cellulose anion exchange chromatography [20]. For infection, C57BL/6 and BALB/c mice were injected intraperitoneally with 10^3^ *T. congolense* VAT TC13 in 100 µl Tris-saline-glucose supplemented with 10% heat-inactivated fetal bovine serum (FBS, Invitrogen, Burlington, ON). In some experiments, BALB/c mice were infected in the footpad (intradermal) with 10^4^ TC13.

### Estimation of parasite burden

At indicated times; a drop of blood from the tail vein was collected onto a glass slide (Fisher Scientific Ottawa, ON) and parasitemia was determined by counting the number of parasites in three or more fields at 40× objective as previously described [21].

### Serum collection and measurement of trypanosome-specific antibodies

At indicated times, mice were anesthetized by intraperitoneal injection of xylazine (10 mg/kg) and ketamine (150 mg/kg) and blood was collected by cardiac puncture using a 1 ml syringe and 25G needle. Blood samples were kept at 4 degree for 4 hr, spun at 2400 rpm for 10 min and, serum was collected and stored at −20°C until used. Serum levels of trypanosome-specific IgM and IgG2a antibodies in infected mice were determined by ELISA as previously described [22].

### Isolation of spleen cells, culture and *ex vivo* Treg staining

At various times after infection, mice were sacrificed and single cell suspensions of the spleens were lysed of contaminating red blood cells (RBC), washed in PBS, counted and resuspended at 4 million/ml in complete medium (DMEM supplemented with 10% heat-inactivated FBS, 2 mM L-glutamine, 100 U/ml penicillin, and 100 µg/ml streptomycin). The cells were plated at 1 ml/well in 24-well tissue culture plates (Falcon, VWR Edmonton, Canada), incubated at 37°C for 72 hr and the culture supernatant fluids were collected and stored at −20°C until assayed for cytokines by ELISA. In some experiments, splenocytes were directly stained *ex vivo* for surface expression of CD3, CD4 and CD25 intracellular expression of Foxp3 using Treg staining kit (BD Bioscience, Mississauga, ON, Canada) according manufacturers suggested protocols.

### Isolation of liver lymphocytes

Intrahepatic lymphocytes were isolated from liver tissues as previously described [23] with minor modifications. Briefly, infected or uninfected mice were anesthetized with isoflourane and blood was collected by cardiac puncture. The chest cavity was opened and the livers were perfused by injecting 10 ml ice-cold PBS into the right ventricle. After 5 min, the liver was minced in collagenase solution (1 mg/ml), digested at 37°C for 1 hour and passed through a 70 µm cell strainer (VWR, ON, Canada). The slurry was washed with 30 ml Hanks balanced salt solution (HBSS) (Invitrogen, ON, Canada) at 1200 rpm for 5 min and the contaminating RBCs were lysed, washed once with HBSS and the cells were resuspended in 4 ml 40% percoll (Sigma). Liver lymphocytes were separated by layering the cells on top of 70% percoll (Sigma) and centrifuging at 750 g at 22°C for 20 min without brakes. The interface containing lymphocytes was carefully collected, washed twice with PBS, re-suspended in complete DMEM medium and stained directly *ex vivo* for cytokines and Tregs as for spleen cells above.

### Cytokine assay

The concentrations of cytokines (IL-6, IL-10, TNF, and IFN-γ) in serum or culture supernatant fluids were assayed by sandwich ELISA using antibody pairs (Ebioscience San Diego, CA) according to the manufacturer suggested instructions. The sensitivities of our ELISA ranges from 7.5–15 pg/ml.

### Depletion of CD25^+^ cells

In some experiments, mice were injected intraperitoneally with 100 µg of anti-CD25 mAb (clone PC61) 24 hrs prior to infection with *T. congolense*. Previous studies from our laboratory have shown that this dose of antibody causes complete and sustained (up to 1 week) depletion of CD25^+^ and FoxP3^+^ cells in *T. congolense*-infected mice [9].

### Isolation and adoptive transfer of CD4^+^CD25^+^ T cells

CD4^+^CD25^−^ and CD4^+^CD25^+^ T cells were isolated from spleen of uninfected C57BL/6 and BALB/c mice by using Stem Cell Treg isolation kit (Stem Cell, Vancouver, BC Canada) according to manufacturers insrtuctions. The purity of CD4^+^CD25^+^ cells was >95% as assessed by flow cytometry. Intracellular staining indicated that isolated CD4^+^CD25^−^ and CD4^+^CD25^+^ cells were ≤5% and ≥ 90% FoxP3^+^, respectively. Groups of mice received four million CD4^+^CD25^−^ or CD4^+^CD25^+^ T cells in PBS by intravenous injection and were infected 24 hrs later with *T. congolense*.

### Statistical analysis

Two-tailed student T test was used to compare mean and standard error of mean (SEM) between two groups. In some other experiments, non-parametric one-way analysis of variance (ANOVA) was used to compare mean and standard deviation (SD) of more than two groups. Tukeys test was used where there was significant difference in ANOVA. Differences were considered significant when p<0.05.

## Results

### Expansion of Tregs in the highly susceptible BALB/c and relatively resistant C57BL/6 mice infected with *T. congolense*

**Figure 1:**
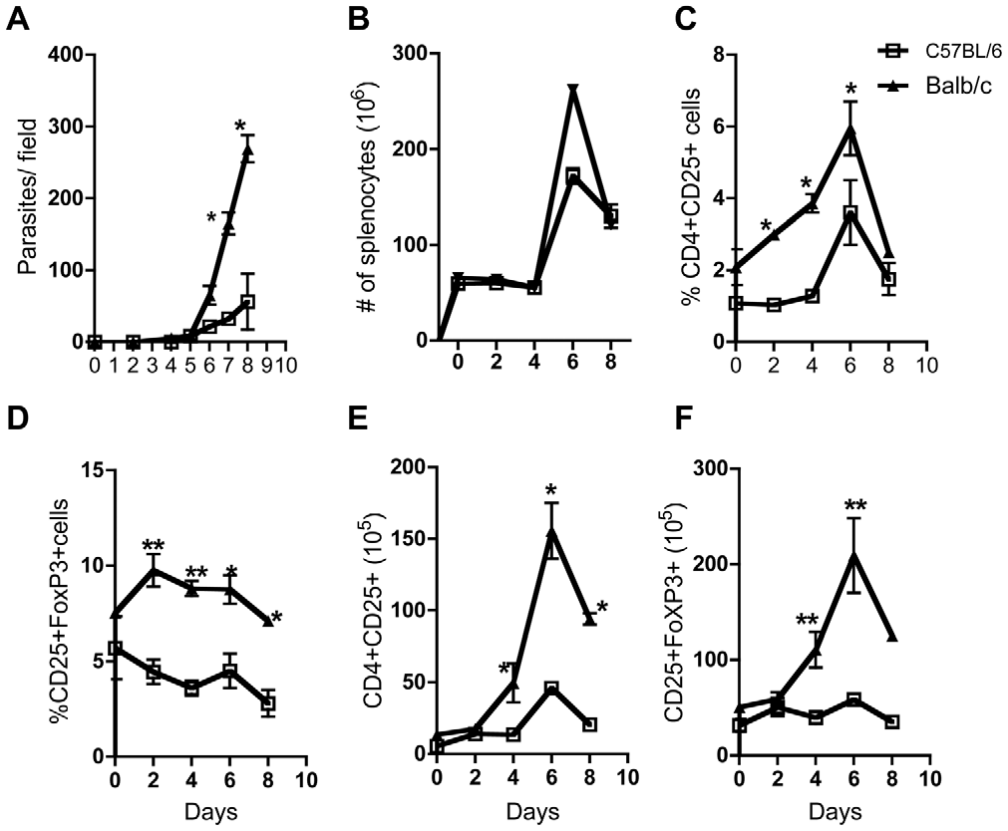
Expansion of Tregs in the highly susceptible and relatively resistant mice infected with *T. congolense*. Female C57BL/6 and BALB/c mice were infected with 10^3^ *T. congolense* (clone TC13) intraperitonealy and parasitemia (A) was monitored daily by counting the number of parasites in a drop of tail blood under ×40 objective magnification. At indicated days, infected mice were sacrificed and the total number of cells (B) in the spleen was determined by trypan blue dye exclusion. Spleen cells were directly stained *ex vivo* and the percentage of CD4^+^CD25^+^ (C) and CD4^+^CD25^+^FoxP3^+^ (D) were determined by flow cytometry. The absolute numbers of CD4^+^CD25^+^ (E) and CD4^+^CD25^+^FoxP3^+^ (F) T cells were determined from the flow cytometric and cell count values. Results presented are representative of 3 independent experiments (n = 3 mice per group) with similar results. (*, p<0.05; **, p<0.01).

Following infection, parasitemia was first detected on day 5 post-infection and was not significantly different in both strains of mice up to day 6 post-infection (Figure 1A). Thereafter, infected BALB/c mice had significantly (p<0.05) higher parasitemia than infected C57BL/6 mice (Figure 1A), consistent with previous report [22]. To determine whether there were differences in expansion of Tregs following *T. congolense* infection, we sacrificed infected mice at days 0 (no infection), 2, 4, 6 and 8 post-infection and determined the total number of cells, the percentages and absolute numbers of CD4^+^CD25^+^FoxP3^+^ cells in the spleens directly *ex vivo* by flow cytometry. There was no significant difference in the total number of cells in the spleens of infected BALB/c and C57BL/6 mice (Figure 1B). Splenocytes from uninfected BALB/c mice had slightly (but not significantly) higher basal numbers of CD4^+^CD25^+^ T cells, which increased steadily after infection compared to infected C57BL/6 mice (p<0.01 Figure 1C & Figure S1A). In addition, uninfected BALB/c mice spleens contain slightly higher basal levels of CD4^+^CD25^+^FoxP3^+^ cells, which transiently increased at days 2 and 4 and dropped to the base line by day 8 post-infection (Figure 1D & Figure S1B). In contrast, the percentage of CD4^+^CD25^+^FoxP3^+^ cells in spleens of infected C57BL/6 mice remained either relatively unchanged or slightly decreased (Figure 1D). Furthermore, the absolute numbers of CD4^+^CD25^+^ and CD4^+^CD25^+^FoxP3^+^ cells in spleens of infected BALB/c mice were higher than those from infected C57BL/6 mice (Figure 1E & F). Collectively, these results show a differential expression and/or expansion of Tregs in the spleens of the highly susceptible and relatively resistant mice after infection with *T. congolense*.

### Differential cytokine profile by splenocytes from *T. congolense*-infected C57BL/6 and BALB/C mice

**Figure 2:**
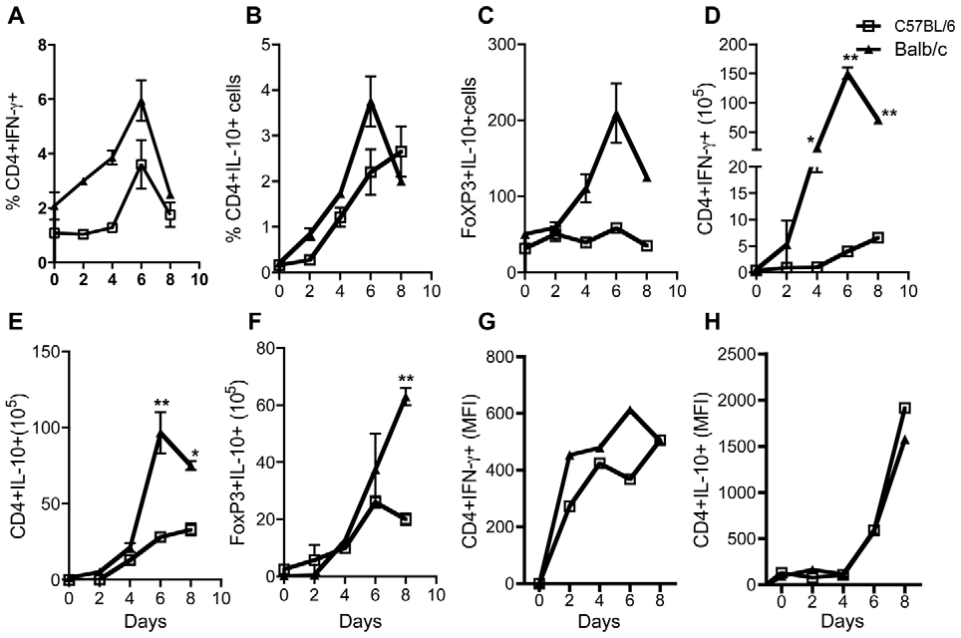
Differential cytokine profile in *T. congolense*-infected C57BL/6 and BALB/c mice. Female C57BL/6 and BALB/c mice infected with *T. congolense* were sacrificed at different time points as indicated and the percentage of CD4^+^IFN-γ ^+^ (A), CD4^+^IL-10^+^ T cells (B), and FoxP3^+^IL-10^+^ (C) T cells were determined directly *ex vivo* by flow cytometry. In addition, the absolute numbers of CD4^+^IFN-γ^+^ (D), CD4^+^IL-10^+^ (E) and FoxP3^+^IL-10^+^ (F), mean flourescence intensity for CD4^+^IFN-γ (G) and CD4^+^IL-10^+^(H) and T cells in the splenocytes were also determined from the flow cytometric and cell count values. Results presented are representative of 3 independent experiments (n = 3–4 mice per group) with similar results. (*, p<0.05; **, p<0.01).

Next, we assessed the immune response to determine how this correlates with the differences in numbers of regulatory T cells in infected C57BL/6 and BALB/c mice. Spleen cells from infected mice were directly stained *ex vivo* for intracellular IFN-γ and IL-10. As shown in Figure 2A & B, there was no significant difference in the percentage of CD4^+^IFN-γ^+^ and CD4^+^IL-10^+^ cells in the spleens of infected BALB/c and C57BL/6 mice although there was a trend towards higher IFN-γ^+^ cells in spleens from infected BALB/c mice (Figure S2).

Furthermore, there was no difference in the percentages of Tregs (CD4^+^CD25^+^Foxp3^+^) that produce IL-10 (Figure 2C). Interestingly, the absolute numbers of CD4^+^IFN-γ^+^ and CD4^+^IL-10^+^ cells were significantly higher in infected BALB/c than in the C57BL/6 mice (Figure 2D & E). In addition, except for day 8 post-infection, there was no significant difference in the absolute numbers of Tregs (CD4^+^CD25^+^Foxp3^+^) that produce IL-10 (Figure 2F) in the two mouse strains.

### Kinetics of Treg expansion and cytokine production in the liver after infection with *T. congolense*

**Figure 3:**
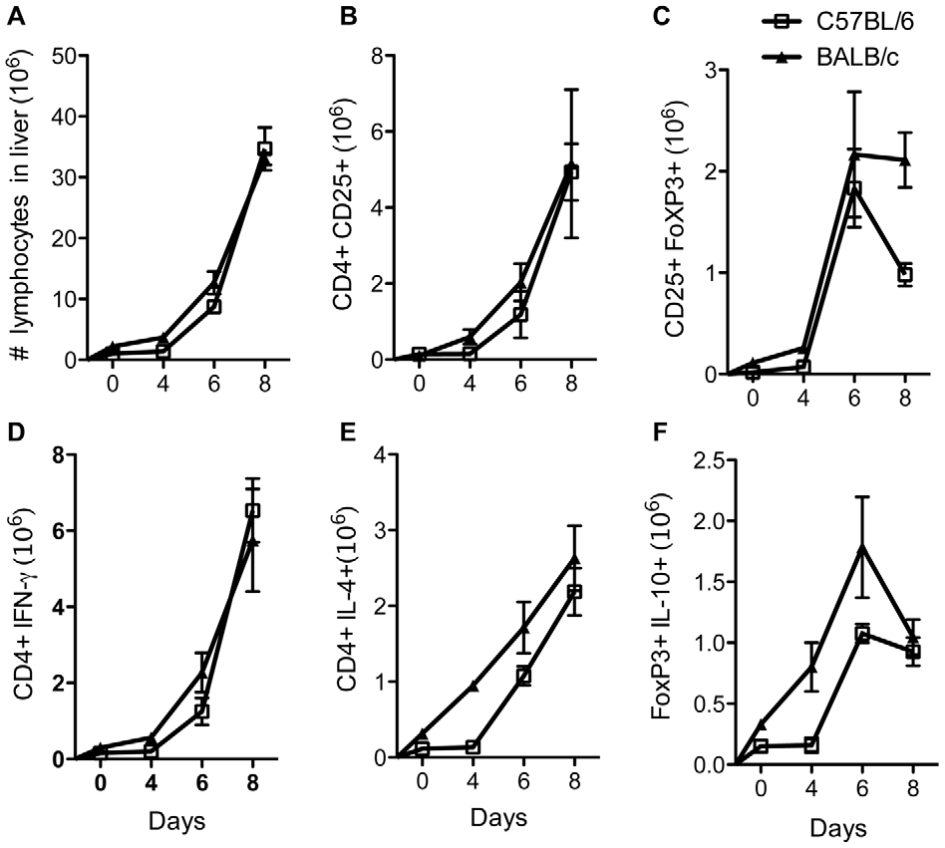
Comparable expansion of Tregs in livers of *T. congolense*-infected BALB/c and C57BL/6 mice. Female C57BL/6 and BALB/c mice were infected with 10^3^ *T. congolense* (clone TC13) intraperitonealy and at indicated days, infected mice were sacrificed and the number of lymphocytes in the liver were determined (A). The absolute number of CD4^+^CD25^+^ (B) and CD4^+^CD25^+^FoxP3^+^ (C) CD4^+^IFN-γ^+^ (D), CD4^+^IL-10**^+^** (E) and FoxP3^+^IL-10^+^ (F) T cells in the liver were determined directly *ex vivo* by flow cytometry. Results presented are representative of 2 independent experiments (n = 3 mice per group) with similar results.

Because liver Tregs have been proposed to contribute to resistance in experimental African Trypanosomiasis [17], we compared the expansion of Tregs and cytokine production by immune cells in the liver of *T. congolense*-infected C57BL/6 and BALB/c mice. As shown in Fig. 3A, although the total number of lymphocytes in the liver increased as the infection progressed, the numbers remain comparable between the two strains of mice. In addition, the absolute numbers of CD25^+^FoxP3^+^ T cells was not different between the two groups until day 8 post-infection when the numbers in infected BALB/c mice were higher (although not significant) than in the C57BL/6 mice (Figure 3B & C). Furthermore, there was also no significant difference in the absolute numbers of CD4^+^IFN-γ^+^, CD4^+^IL-10^+^ and FoxP3^+^IL-10^+^ T cells (Figure 3D–F) in the livers of infected mice of both strains of mice. Overall, the data presented here show that unlike the spleen, the expansion of Tregs in the liver was comparable between BALB/c and C57BL/6 mice during infection with *T. congolense*.

### Depletion of CD25^+^ cells enhances resistance to experimental *Trypanosoma congolense* infection

**Figure 4:**
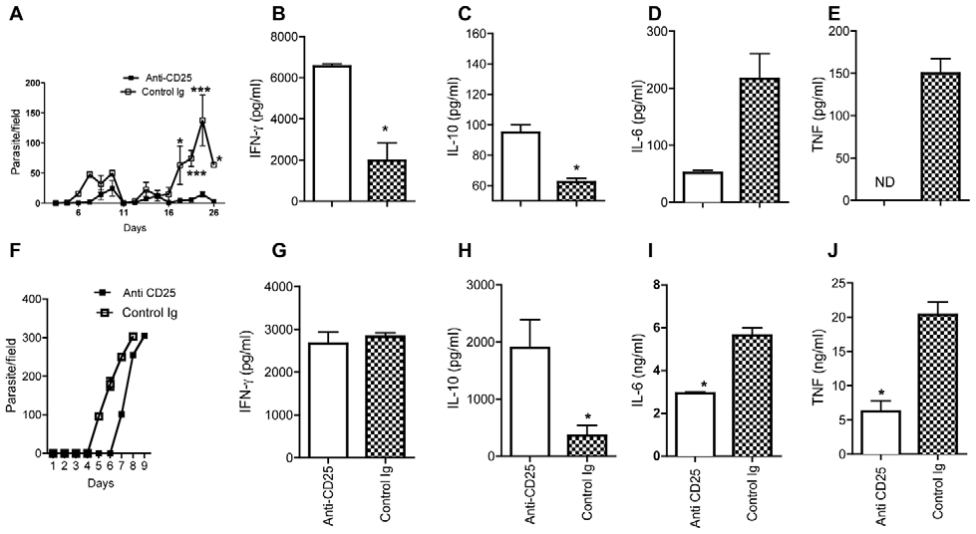
Depletion of CD25^+^ T cells enhances resistance to experimental *T. congolense* infection. C57BL/6 and BALB/c mice were injected with anti-CD25 mAb (100 µg to deplete CD25^+^ cells) or control Ig. After 24 hrs, mice were infected with *T. congolense* and the number of parasites in the blood was determined over the indicated time period (A and F). At 8 (BALB/c, G and H) and 26 (C57BL/6, B–E) days after infection, mice were sacrificed and the serum levels of IFN-γ (B and G), IL-10 (C and H), IL-6 (D and I) and TNF (E and J) was determined by ELISA. Results presented are representative of 2 independent experiments (n = 5–6 mice per group) with similar results. (*, p<0.05; **, p<0.01; *** p<0.001).

To determine whether Tregs play a differential role in the pathogenesis of experimental *T. congolense* infection in BALB/c and C57BL/6 mice, we injected BALB/c and C57BL/6 mice with anti-CD25 monoclonal antibody one day prior to infection with *T. congolense*, followed by weekly injection in infected C57BL/6 mice for the next 2 weeks. Although anti-CD25 mAb could also potentially deplete all CD25^+^ cells, it preferentially caused sustained depletion of CD4^+^Foxp3^+^ cells (Tregs) throughout the treatment period [9]. Depletion of Tregs resulted in longer prepatent period (not statistically significant) and significantly (p<−0.05–0.001) lower first and second peaks of parasitemia (Figure 4A) but did not affect the survival period of infected C57BL/6 mice (data not shown). In addition, serum levels of IL-10 and IFN-γ; cytokines that have been shown to regulate resistance to *T. congolense* infection [9], [21], [22], [24], as well the percentages of cells producing these cytokines in the spleens (Figure S3A & B) were significantly (p<0.05) higher in Tregs-depleted mice compared to those treated with isotype control (Figure 4B & C), suggesting that anti-CD25 mAb treatment did not negatively impact on effector Th1 (IFN-γ-producing) cells. Paradoxically, depletion of Tregs led to a significant reduction in serum levels of IL-6 (Figure 4D) and TNF (Figure 4E) in infected mice. Interestingly, depletion of Tregs also caused a 2-day delay in onset of parasitemia but did not significantly affect the peak parasitemia (Figure 4F), survival period (data not shown) and serum levels of IFN-γ (Figure 4G) in infected BALB/c mice. However, while the serum IL-10 level was significantly (p<0.01) increased in Treg-depleted mice, serum levels of IL-6 and TNF (Figure 4 I & J) were significantly decreased when compared to control Ig.

**Figure 5:**
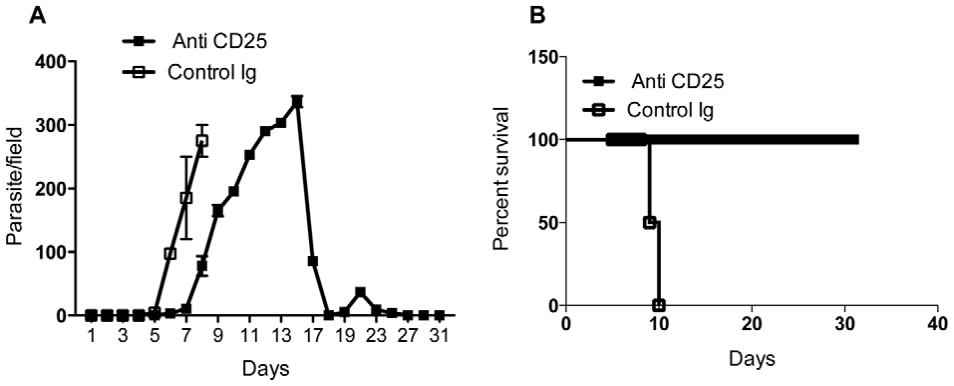
The effect of anti-CD25 mAb treatment in infected BALB/c mice is dependent on infection route. BALB/c mice were injected intraperitoneally with 100 µg of anti-CD25 mAb (PC61) or control Ig. After 24 hr, mice were infected with 10^3^ *T. congolense* clone TC13 in the footpad and parasitemia (A) and survival period (B) were determined until the experiment was discontinued. Results presented are representative of 2 independent experiments (n = 4–6 mice per group) with similar results.

Because the previous report that showed anti-CD25 mAb treatment enhanced resistance in BALB/c mice utilized intradermal infection route [18], we wondered whether the marginal effect we observed was related to the use intraperitoneal route, which causes rapid and uncontrolled infection in BALB/c mice [25]. Therefore we repeated the antibody treatment experiment but infected mice with TC13 in the footpad. As shown in Figure 5A & B, 100% of Treg-depleted mice infected with *T. congolense* in the footpad controlled their first wave of parasitemia and survived up to 21 days when the experiment was terminated. We conclude that as in C57BL/6 mice, naturally occurring regulatory T cells contribute to susceptibility in BALB/c mice and this effect is more apparent following intradermal infection.

### Adoptive transfer of CD4^+^CD25^+^ T cells leads to increased parasitemia in the relatively resistant C57BL/6 mice

**Figure 6:**
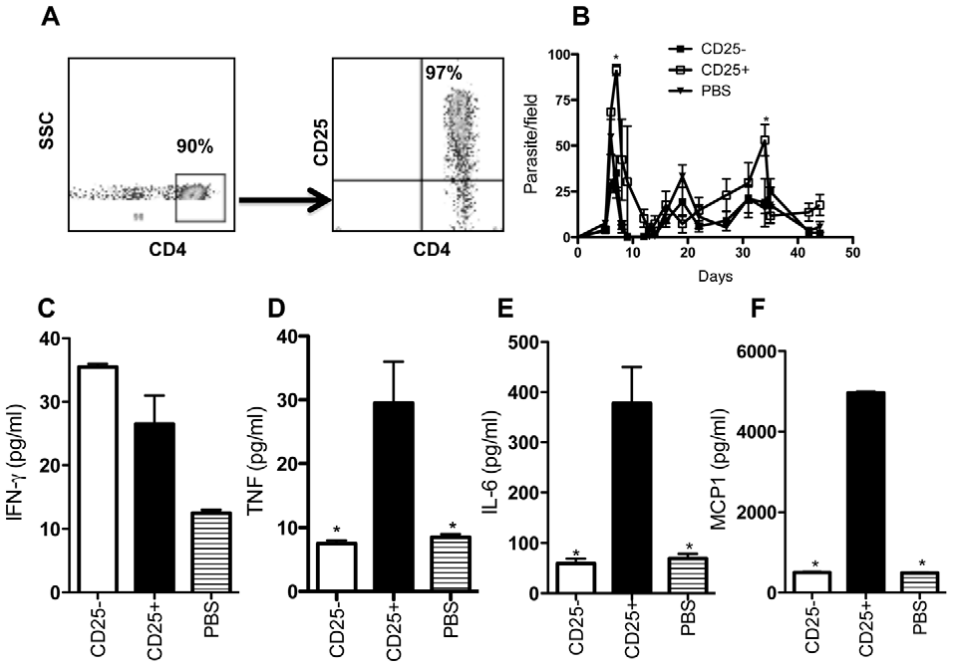
Adoptive transfer of Tregs increases parasitemia and serum proinflammatory cytokine levels in C57BL/6 mice. Four million highly enriched (>95%) CD4^+^CD25^+^ or CD4^+^CD25^−^ T cells (A) isolated from naïve C57BL/6 mice were transferred (by intravenous route) into naïve recipient mice that were subsequently infected 24 hr later with 10^3^ *T. congolense* clone TC13. Parasitemia was determined daily for up to 44 days post infection (B). Some mice were sacrificed at day 13 post-infection and serum levels of IFN-γ (C), TNF (D), IL-6 (E), and MCP-1 (F) was measured by ELISA. Results presented are representative of 2 independent experiments with similar findings (*, p<0.05).

To more clearly determine the role of Tregs in the pathogenesis of experimental African trypanosomiasis, we adoptively transferred highly enriched (>97%, Fig. 6A) naïve syngeneic CD4^+^CD25^+^ (Tregs) and CD4^+^CD25^−^ (non Tregs) cells (4 million cells/mouse) into naïve C57BL/6 mice and after 24 hr infected them with *T. congolense*. As shown in Figure 6B, recipients of CD4^+^CD25^+^ T cells had higher peak parasitemia and took longer time to control their first wave of parasitemia compared to recipients of CD4^+^CD25^−^ cells or PBS. In addition, while there was no significant difference in serum IFN-γ levels among the groups (Figure 6C), recipients of CD4^+^CD25^+^ T cells had significantly higher levels of proinflammatory cytokines (including TNF, IL-6 and MCP-1) than those that received CD4^+^CD25^−^ or PBS on day 13 post-infection (Figure 6D–F). Interestingly, this difference in serum levels of proinflammatory cytokines was transient, such that when mice were sacrificed at day 21 post-infection (a time when the difference in parasitemia was no longer significant), this difference in serum levels of TNF, MCP-1 and IL-6 between recipient and PBS control groups was no longer apparent (data not shown). Interestingly, adoptive transfer of CD4^+^CD25^+^ T cells also caused a significant reduction in serum levels of trypnosome-specific IgM and IgG2a in infected mice (Figure S4A & B). Collectively, these results suggest that naturally occurring Tregs suppress efficient early parasite control in the relatively resistant C57BL/6 mice infected with *T. congolense*.

## Discussion

In this study, we investigated the role of regulatory T cells in pathogenesis of experimental *T. congolense* infection in mice. Our findings indicate that naturally occurring regulatory T cells contribute to enhanced disease in both the relatively resistant (C57BL/6) and highly susceptible (BALB/c) strains of mice. Following *T. congolense* infection of BALB/c mice, the percentage and absolute numbers of splenic Tregs steadily increased as the infection progressed. This was in sharp contrast to the relatively unchanged numbers of these cells in the infected C57BL/6 mice. Interestingly, there was no difference in the expansion of Tregs in the liver of infected BALB/c and C57BL/6 mice, suggesting that differences in hepatic Tregs do not contribute to the differences in the outcome of *T. congolense* infection in these mice. Depletion studies showed that Tregs negatively affect efficient parasite control while adoptive transfer studies confirmed the role of these cells in control of parasitemia and production of disease exacerbating proinflammatory cytokines.

Unlike cutaneous leishmaniasis, where the role of regulatory T cells have been well characterized both in primary [26] and secondary [16] immunity, the role of these cells in pathogenesis of experimental *T. congolense* infection is still unclear. While some reports suggest that Tregs enhance susceptibility [18], others indicate that they are protective [27]. Although, both studies used *T. congolense* clone TC13 (as in our study) for infection, they used different strains of mice and different dose of anti-CD25 mAb to deplete CD25^+^ T cells. We have studied the role of Tregs in *T. congolense* infection in both the susceptible and relatively resistance strains of mice under identical experimental conditions. Collectively, our depletion and adoptive transfer studies show that Tregs contribute in part to impaired parasite control (BALB/c and C57BL/6) and early death (BALB/c) in experimental *T. congolense* infection. Thus, our results support the findings of Wei et al [27] and show that Tregs play a pathogenic role in experimental African trypanosomiasis independent of mouse genetic background.

Because some studies have shown that anti-CD25 mAb is not very specific at depleting only Tregs, [28], we also used adoptive transfer experiments to further define the role of Tregs in experimental *T. congolense* infection cells. We showed that adoptive transfer of highly enriched naïve CD4^+^CD25^+^ T cells (Tregs) led to increased peak parasitemia and production of disease exacerbating inflammatory cytokines, including IFN-γ, IL-6, TNF and MCP-1 early during infection in C57BL/6 mice (Figure 4 A–E). Interestingly, adoptive transfer of Tregs did not alter parasitemia or serum levels of proinflammatory cytokines in the BALB/c mice, most likely because these mice are already highly sensitive and succumb rapidly to intraperitoneal infection with *T. congolense*. The finding that depletion and/or adoptive transfer of Tregs decreases and increases the production of proinflammatory cytokines, respectively, is paradoxical because Tregs are normally known to dampen inflammatory responses [29]. However, recent reports have shown that Tregs could also enhance CD4^+^ T cell-mediated inflammatory lung fibrosis [30] and IL-6 production by mast cells [31]. Thus, it is conceivable that as in these reports, Tregs could enhance inflammation in experimental *T. congolense* infection although the exact mechanism(s) remains to be elucidated.

How does Tregs contribute to enhanced susceptibility to experimental African trypanosome infection? Previous reports suggest that production of IL-10 by Tregs might be critically important in dampening excessive inflammatory response leading to enhanced survival in *T. congolense* infected mice [32]. However, we found no significant difference in the percentage and absolute numbers of IL-10-producing Tregs (CD4^+^FoxP3^+^IL-10^+^) cells in the spleens and livers of infected BALB/c and C57BL/6 mice. Thus, our results do not support the proposal that Treg-derived IL-10 contributes to enhanced-resistance to experimental African trypanosomiasis. We speculate that these cells might act by suppressing the production of IFN-γ and IL-10 by helper T cells or other as yet uncharacterized cells that contribute to efficient parasite clearance and enhanced resistance in infected mice. In line with this, we found that depletion of CD25^+^ T cells by anti-CD25 mAb led to a significant increase in serum levels of IFN-γ and IL-10, and this was associated with increased prepatent period and lower parasitemia in infected mice (Figure 3A–C). Another possible mechanism by which Tregs may enhance susceptibility to experimental *T. congolense* infection is by suppressing antibody response. Tregs have been shown to directly or indirectly (via their effect on CD4^+^ helper T cells) suppress B cell responses leading to impaired antibody production [33], [34]. Since antibodies are critically important for controlling parasitemia and resistance to African trypanosomes, a suppressive effect on their production will result in enhanced susceptibility to the disease. In line with this, we found that adoptive transfer of Tregs was associated with a significant reduction in serum levels of anti-*T. congolense* IgM and IgG2a antibodies in infected mice (Figure S4A & B).

Generalized immunosuppression is a prominent feature of both natural (cattle) and experimental (mice) African trypanosomiasis [35], [36], [37]. However, the exact mechanism through which African trypanosomes induce immunosuppression is still not well defined. While earlier studies implicated macrophages [36] others suggest that T cells may be involved in this process [38], [39]. In an exhaustive review, Tabel et al [32] suggested that Tregs maybe involved in immunosuppression via the production of IL-10, which down-regulates and/or inhibits IFN-γ and nitric oxide production [21]. In our study, we found no difference in percentages and absolute numbers of IL-10-producing CD4^+^FoxP3^+^ cells in infected highly susceptible and relatively resistant mice. On the contrary, depletion of Tregs (by anti-CD25 mAb treatment) was associated with increased IFN-γ and IL-10 serum levels and increased frequency of IFN-γ and IL-10-producing CD4^+^ T cells in infected mice. IFN-γ and IL-10 play critical roles in resistance to experimental *T. congolense* infection in mice [9], [40], [41]. Thus, our studies suggest that IL-10-derived Tregs may not be involved in susceptibility to *T. congolense* infection. They suggest that the effects of Tregs maybe directly related to their suppression of IFN-γ and antibody responses, leading to failure to control parasitemia.

Several studies have shown that co-production of IL-10 by IFN-γ producing CD4^+^ Th1 cells is critically important for regulation of excessive Th1 and inflammatory responses in many infections [42]. In experimental *T. gondii* infection, Jankovic et al [43] demonstrated that IL-10 producing CD4^+^T-bet^+^ Th1 cells (and not Foxp3^+^ T cells) are the major regulators of inflammation in infected mice. Similarly, Anderson et al [44] showed in a model of chronic cutaneous leishmaniasis caused by *L. major* that in addition to CD25^+^Foxp3^+^ Treg cells, IFN-γ-producing CD4^+^CD25^−^Foxp3^−^ (Th1 cells) that co-produce IL-10 are more important than Tregs at regulating inflammation and disease chronicity. In this study, we carefully analyzed our data to determine whether there were CD4^+^IFN-γ^+^IL-10^+^ (double producers) T cells but failed to observe any significant number of such cells in the spleens or livers of infected mice (Figure S2). Therefore, it is unlikely that these so called “Th1 regulatory effector cells” play a significant role in immunoregulation in experimental *T. congolense* infection in mice.

In conclusion, we have shown that there are differences in the kinetics and relative expansion of Tregs following *T. congolense* infection in both the highly susceptible and relatively resistant mice. Our results show that Tregs contribute to enhanced susceptibility in experimental murine trypanosomiasis in both the highly susceptible and relatively resistant mice.

## Supporting Information

Figure S1 **Representative dot plots of Tregs and IL-10-producing cells in the spleens of infected mice.** Female BALB/c and C57BL/6 mice infected with *T. congolense* were sacrificed at day 0 (upper panel) and day 6 (lower panel) and the percentage of CD4^+^CD25^+^ (A), FoxP3^+^IL-10^+^ (B), and FoxP3^+^CD25^+^ (C) T cells were determined directly *ex vivo* by flow cytometry.
(TIF)

Figure S2 **Representative dot plots of CD4^+^IFN-γ^+^IL-10^+^ cells in the spleens of infected mice.** Female BALB/c and C57BL/6 mice infected with *T. congolense* were sacrificed at different time points as indicated and the percentage of IL-10^+^IFN-γ^+^ T cells within CD4^+^ T cell population were determined directly *ex vivo* by flow cytometry.
(TIF)

Figure S3 **Treatment with anti-CD25 mAb increases the percentages of splenic CD4^+^IFN-γ and CD4^+^IL-10^+^ cells.** Female BALB/c and C57BL/6 mice were injected with anti-CD25 mAb (100 µg to deplete CD25^+^ cells) or control-Ig. After 24 hrs, mice were infected with *T. congolense*. At day 8 (BALB/c, A) and day 13 (C57BL/6, B) post-infection, mice were sacrificed and the percentage of CD4^+^IFN-γ (upper panel) CD4^+^IL-10^+^ mice (lower panel) were determined directly *ex vivo* by flow cytometry.
(TIF)

Figure S4 **Adoptive transfer of CD4^+^CD25^+^ T cells leads to suppression of** ***Trypanosome*** **-specific IgM and IgG2a antibodies.** Four million CD4^+^CD25^+^ or CD4^+^CD25^−^ T cells were isolated from spleens of naïve C57BL/6 mice and transferred into naïve recipient that were subsequently infected with *T. congolense* after 24 hrs. Recipient mice were sacrificed on day 13 and serum levels of *T. congolense*-specific IgM (A) and IgG2a (B) were measured by ELISA. (*, p<0.05; **, p<0.01).
(TIF)
