# Supplementary figures and images for: Regulatory T Cells Enhance Susceptibility to Experimental Trypanosoma Congolense Infection Independent of Mouse Genetic Background
Source: PLoS Negl Trop Dis. 2012 Jul 31;6(7):e1761. doi: 10.1371/journal.pntd.0001761 (PMC3409116; doi:10.1371/journal.pntd.0001761)

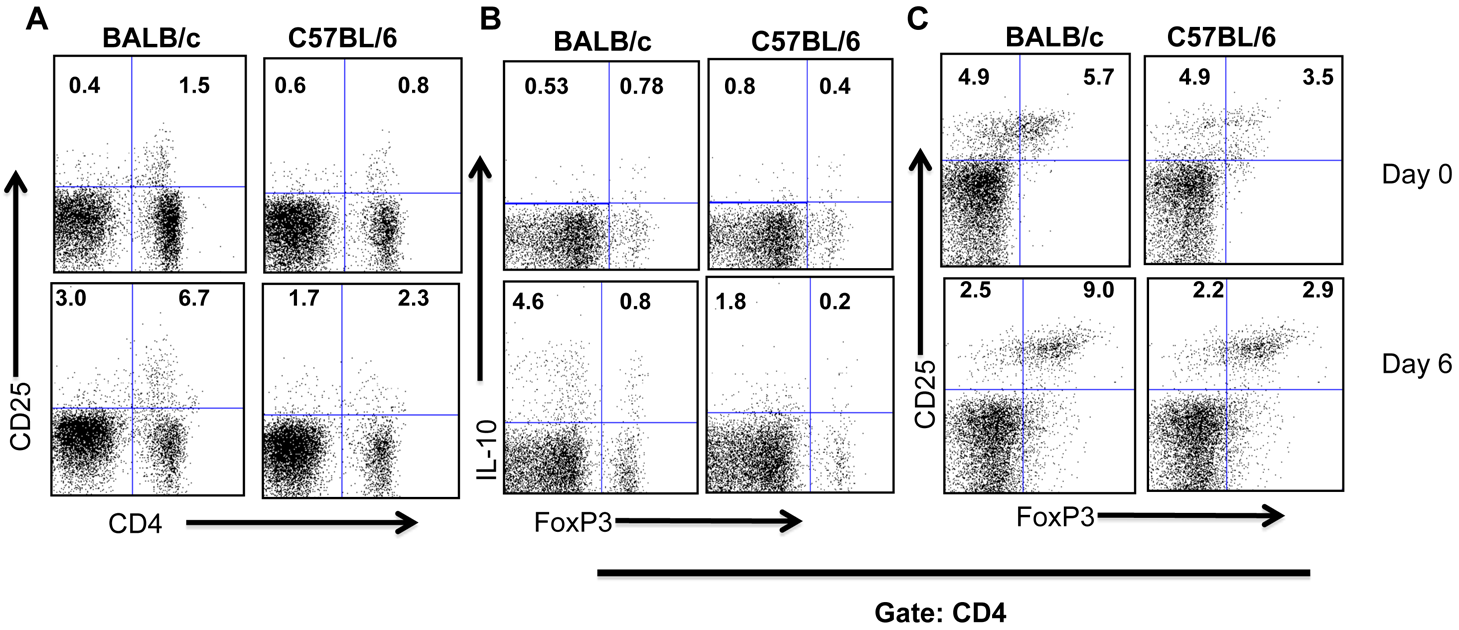

Supplement: Figure S1 — Representative dot plots of Tregs and IL-10-producing cells in the spleens of infected mice. Female BALB/c and C57BL/6 mice infected with T. congolense were sacrificed at day 0 (upper panel) and day 6 (lower panel) and the percentage of CD4+CD25+ (A), FoxP3+IL-10+ (B), and FoxP3+CD25+ (C) T cells were determined directly ex vivo by flow cytometry. (TIF) [file pntd.0001761.s001.tif]

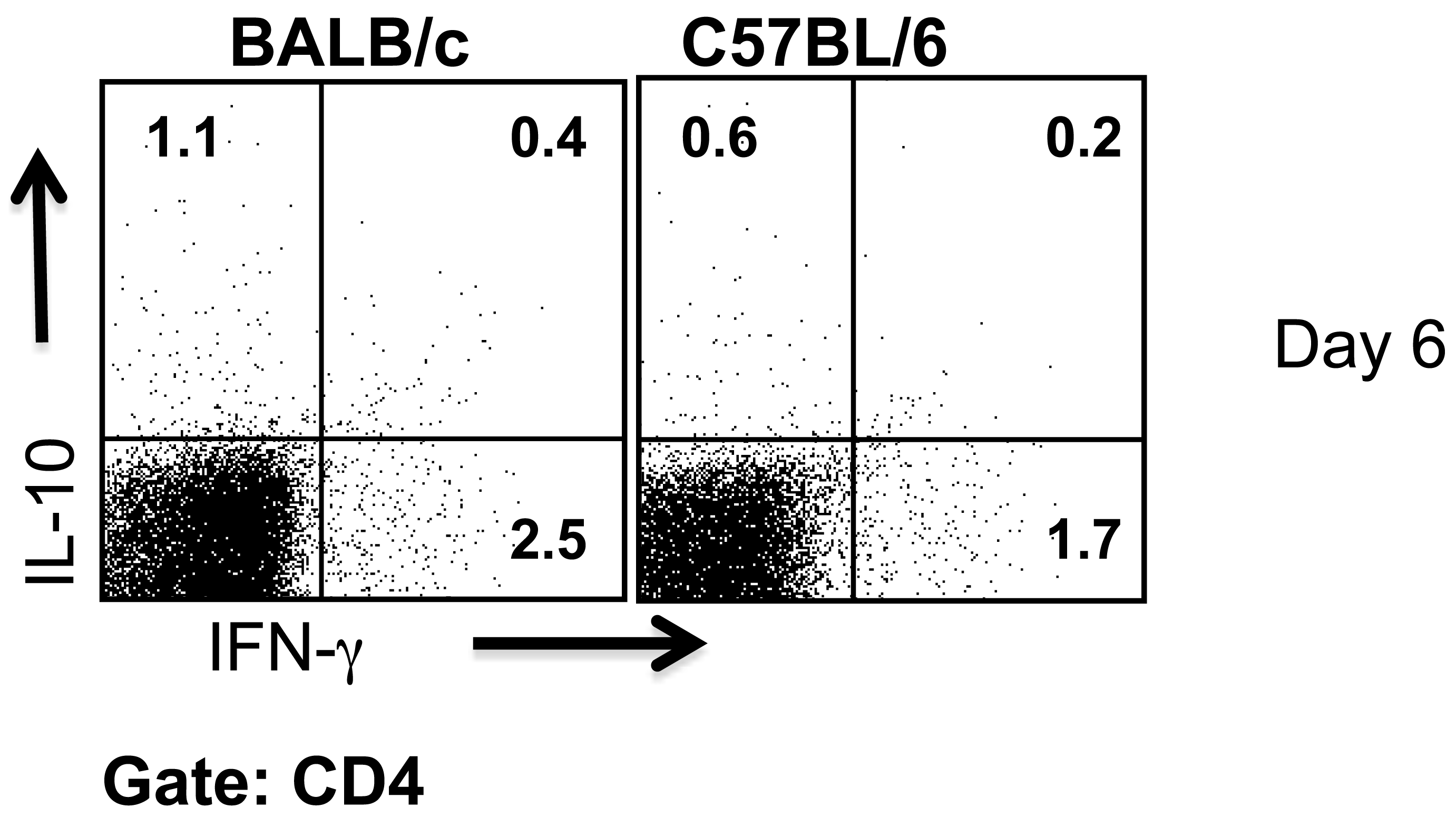

Supplement: Figure S2 — Representative dot plots of CD4+IFN-γ+IL-10+ cells in the spleens of infected mice. Female BALB/c and C57BL/6 mice infected with T. congolense were sacrificed at different time points as indicated and the percentage of IL-10+IFN-γ+ T cells within CD4+ T cell population were determined directly ex vivo by flow cytometry. (TIF) [file pntd.0001761.s002.tif]

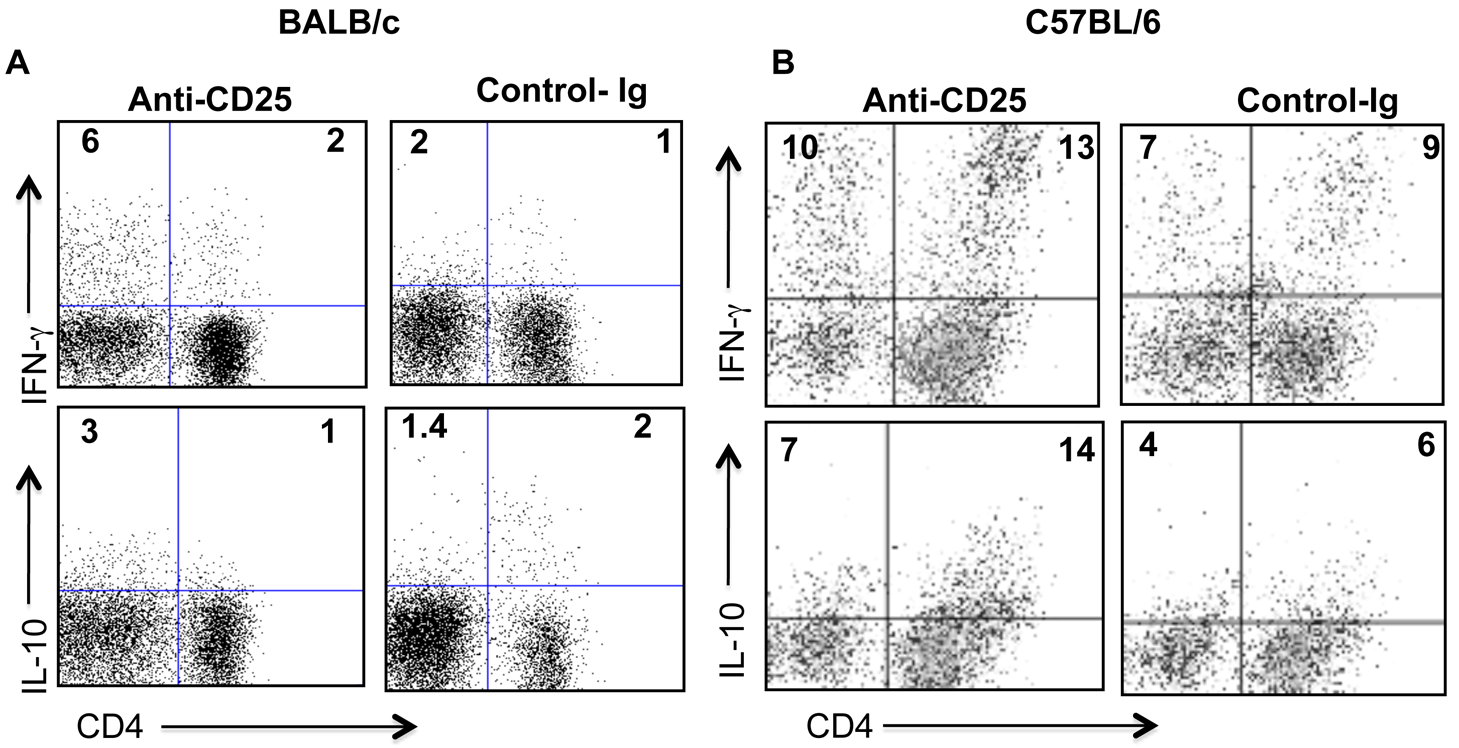

Supplement: Figure S3 — Treatment with anti-CD25 mAb increases the percentages of splenic CD4+IFN-γ and CD4+IL-10+ cells. Female BALB/c and C57BL/6 mice were injected with anti-CD25 mAb (100 µg to deplete CD25+ cells) or control-Ig. After 24 hrs, mice were infected with T. congolense. At day 8 (BALB/c, A) and day 13 (C57BL/6, B) post-infection, mice were sacrificed and the percentage of CD4+IFN-γ (upper panel) CD4+IL-10+ mice (lower panel) were determined directly ex vivo by flow cytometry. (TIF) [file pntd.0001761.s003.tif]

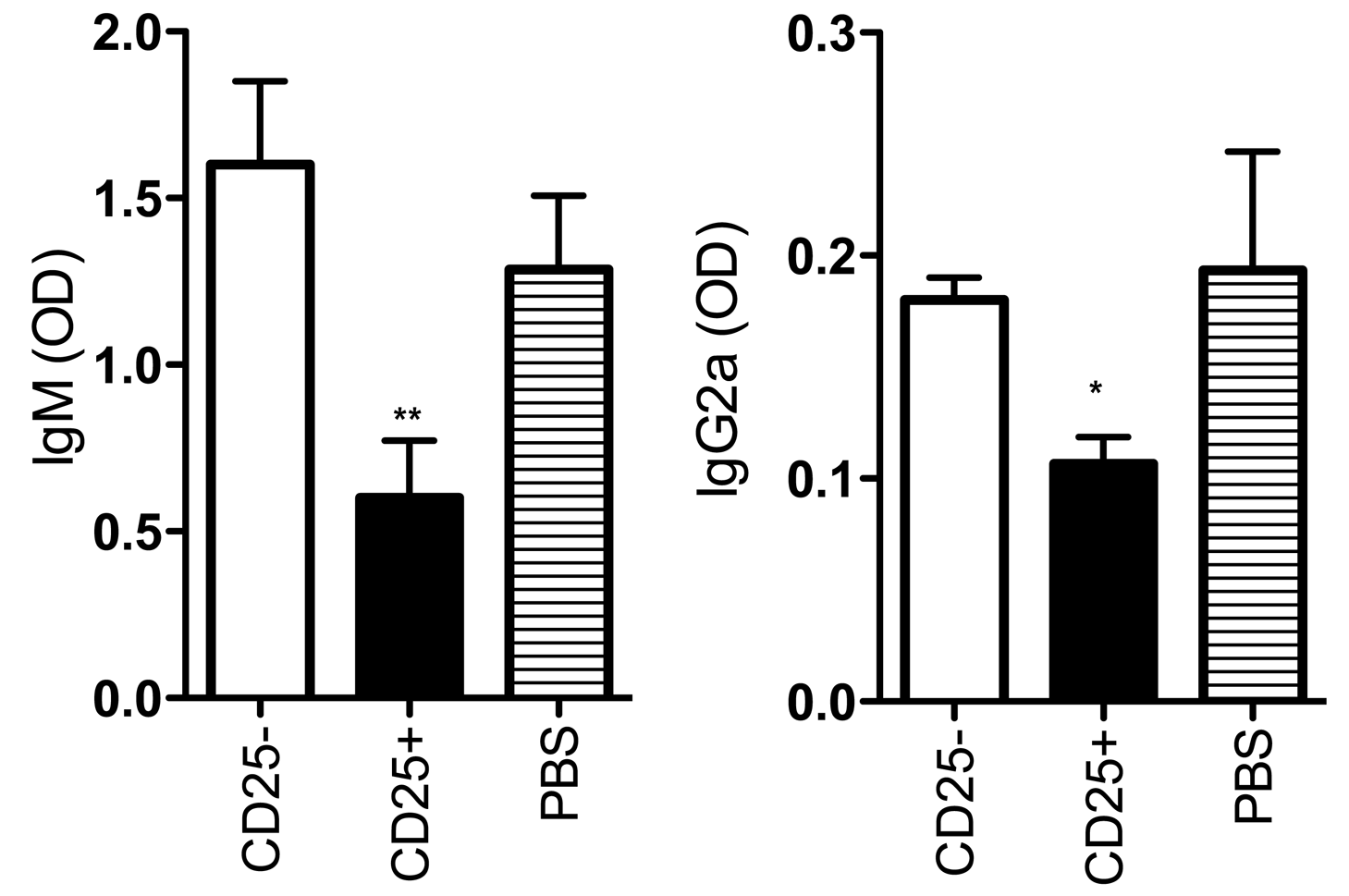

Supplement: Figure S4 — Adoptive transfer of CD4+CD25+ T cells leads to suppression of Trypanosome -specific IgM and IgG2a antibodies. Four million CD4+CD25+ or CD4+CD25− T cells were isolated from spleens of naïve C57BL/6 mice and transferred into naïve recipient that were subsequently infected with T. congolense after 24 hrs. Recipient mice were sacrificed on day 13 and serum levels of T. congolense-specific IgM (A) and IgG2a (B) were measured by ELISA. (*, p<0.05; **, p<0.01). (TIF) [file pntd.0001761.s004.tif]
